# Expanding the population coverage of evidence–based interventions with community health workers to save the lives of mothers and children: an analysis of potential global impact using the Lives Saved Tool (LiST)

**DOI:** 10.7189/jogh.07.020401

**Published:** 2017-12

**Authors:** Victoria B Chou, Ingrid K Friberg, Mervyn Christian, Neff Walker, Henry B Perry

**Affiliations:** 1 Institute for International Programs, Johns Hopkins Bloomberg School of Public Health, Baltimore, Maryland, USA; 2 Norwegian Institute of Public Health, Oslo, Norway; 3 Johns Hopkins Bloomberg School of Public Health, Baltimore, Maryland, USA

## Abstract

**Background:**

Evidence has been accumulating that community health workers (CHWs) providing evidence–based interventions as part of community–based primary health care (CBPHC) can lead to reductions in maternal, neonatal and child mortality. However, investments to strengthen and scale–up CHW programs still remain modest.

**Methods:**

We used the Lives Saved Tool (LiST) to estimate the number of maternal, neonatal and child deaths and stillbirths that could be prevented if 73 countries effectively scaled up the population coverage of 30 evidence–based interventions that CHWs can deliver in these high–burden countries. We set population coverage targets at 50%, 70%, and 90% and summed the country–level results by region and by all 73 high–burden countries combined. We also estimated which specific interventions would save the most lives.

**Findings:**

LiST estimates that a total of 3.0 (sensitivity bounds 1.8–4.0), 4.9 (3.1–6.3) and 6.9 (3.7–8.7) million deaths would be prevented between 2016 and 2020 if CBPHC is gradually scaled up during this period and if coverage of key interventions reaches 50%, 70%, and 90% respectively. There would be 14%, 23%, and 32% fewer deaths in the final year compared to a scenario assuming no intervention coverage scale up. The Africa Region would receive the most benefit by far: 58% of the lives saved at 90% coverage would be in this region. The interventions contributing the greatest impact are nutritional interventions during pregnancy, treatment of malaria with artemisinin compounds, oral rehydration solution for childhood diarrhea, hand washing with soap, and oral antibiotics for pneumonia.

**Conclusions:**

Scaling up CHW programming to increase population–level coverage of life–saving interventions represents a very promising strategy to achieve universal health coverage and end preventable maternal and child deaths by 2030. Numerous practical challenges must be overcome, but there is no better alternative at present. Expanding the coverage of key interventions for maternal nutrition and treatment of childhood illnesses, in particular, may produce the greatest gains. Recognizing the millions of lives of mothers and their young offspring that could be achieved by expanding coverage of evidence–based interventions provided by CHWs and strengthening the CBPHC systems that support them underscores the pressing need for commitment from governments and donors over the next 15 years to prioritize funding, so that robust CHW platforms can be refined, strengthened, and expanded.

The launch of the new Sustainable Development Goals (SDGs) in 2015 [1] gives stakeholders in global health a unique opportunity to acknowledge notable progress, assess current conditions, and designate future priorities. Monitoring of country–specific mortality trends indicates that only a minority of the world’s low– and middle–income countries ultimately reached designated Millennium Development Goal (MDG) targets for either MDG 4 (to reduce mortality of children younger than 5 years of age) or MDG 5 (to reduce maternal mortality) [2]. Furthermore, among the Countdown to 2015 (Countdown) priority countries [2], median population coverage for a third of the 21 high–impact maternal and child health interventions remains less than 50%, with notably low coverage of interventions around the time of birth and for managing childhood infection [2].

Devising better approaches to expand coverage of key evidence–based interventions is clearly needed considering that each year 5.9 million deaths still occur among under–5 children [3], 289 000 deaths among women from maternal causes [4], and 2.6 million stillbirths [5], with a majority of these deaths due to preventable or treatable conditions. Accelerating the global rate of reduction of maternal, neonatal and child mortality to end preventable child and maternal deaths remains one of the great unfinished global health agendas of our era. Achieving this will require effective scaling–up so progress can be made toward “universal access to quality essential health–care services,” one of the central SDG3 health targets guiding global health development initiatives over the next 15 years [6].

Over the past two decades, evidence has been steadily accumulating that community health workers (CHWs) can play an essential role to deliver many evidence–based interventions for improving maternal, neonatal, and child health (MNCH) [7,8]. CHWs are paraprofessionals or lay individuals from the community, are familiar with the local context, and likely have an in–depth understanding of the indigenous culture. CHWs have typically received a standard training on specific topics of shorter duration than health professionals and they work within their village posts at household and community level to promote healthy behaviors and provide basic services [9]. CHWs and community–based services are increasingly gaining recognition as valuable contributors, because they can effectively expand access to and delivery of health services as well as promote healthy household behaviors, particularly for mothers and children [10]. In addition to direct delivery or provision of services, CHWs can play an essential role by increasing demand for services, serve as key linkages to facility–based interventions, or raise health awareness via promotion or advocacy in communities with limited resources [7]. Many Countdown countries with notable progress for MDGs 4 and 5 have strong national CHW programs [8]. CHW programs may be comprised of health workers acting as volunteers or paid civil servants, or in a capacity somewhere in between these two extremes. Mobilizing a strong and effective health workforce to strengthen health systems and accelerate gains toward universal health coverage was highlighted as a vital priority by the World Health Assembly in 2016 [11].

In this paper, we estimate the impact of MNCH outcomes, if CHWs were able to expand the population coverage of evidence–based interventions across the continuum of care through community–based programming. Our analysis estimates the numbers of deaths that could be prevented in 73 Countdown countries if coverage of these life–saving MNCH interventions were expanded by CHW cadres as a result of robust capacity building and enhanced training to complement services provided at facilities.

## METHODS

**Figure 1:**
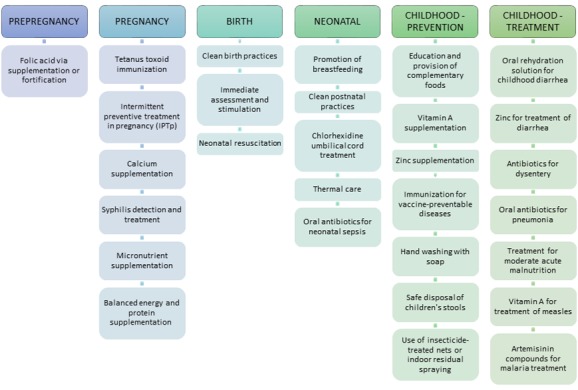
Evidence–based interventions that can be provided by community health workers that have been included in the Lives Saved Tool calculations.

We identified evidence–based interventions included in the community platform (excluding sexual and reproductive health interventions) as defined for the *Disease Control Priorities Volume 2* [12]. The community platform consists of all evidence–based interventions that can be delivered by locally based CHWs or by outreach CHWs for child health days when immunizations, vitamin A, and other interventions are given. Evidence to support the effectiveness of these interventions is available elsewhere [7,13]. As shown in **Figure 1**, the listed interventions span across a continuum of care from pre–pregnancy care to the prevention and treatment of childhood diseases. Our theory of change assumes that adequate numbers of CHWs could be trained and supported in order to effectively deliver these key interventions in the community. We did not estimate the potential benefit of other activities often carried out by CHWs, such as promoting utilization of health care facilities, educating about family planning or other healthy behaviors, and fostering empowerment.

We used the Lives Saved Tool (LiST), a data–driven modeling platform, to estimate the number of lives that could be saved by reducing the risk of death through the expansion of CHW–led population coverage of specific evidence–based health interventions [14].

Details about the LiST methodology have been described elsewhere [15]. Briefly, LiST estimates country–specific maternal, child, and pregnancy outcomes based upon changes in population–level coverage of interventions while taking into account that country’s underlying health status, cause–specific mortality distribution, and the best available estimates of intervention effectiveness using a linear deterministic model. As a module within the Spectrum package, LiST has linkages for parameters including demography, fertility determinants, and HIV/AIDS interventions. Data about population–level coverage for each intervention, defined ideally as the proportion of women and children in need of life–saving intervention who actually receive it [16], are abstracted from the most recent nationally–representative household surveys and sources included the Demographic and Health Surveys (DHS), Multiple Indicator Cluster Surveys (MICS), and other nationally–representative household surveys.

LiST was applied at the country level to calculate the number of deaths that would be averted in Countdown countries if coverage of each CHW–provided intervention was expanded to reach at least 50%, 70% or 90% in the target population. Mexico and China were excluded from the analysis due to limited data about intervention coverage and a low under–five mortality rate (<15 deaths per 1000 live births). The final sample included the remaining 73 Countdown countries. Version 5.43 beta19 of LiST was used for all analyses.

Each country was analyzed separately to examine three scenarios of scale up (50%, 70% or 90% targets) for the subset of MNCH interventions in the community platform. The counterfactual was a baseline model for each country which assumed no change in health intervention coverage between 2015 and the end year of 2020. Projected changes in fertility, HIV/AIDS, and demography were available based upon the Spectrum defaults for secular trends. A pattern of linear increase was modeled to reach designated targets by the end year of 2020. If coverage of an intervention was already reported to be equivalent or above the designated coverage target, coverage was maintained at its current level. The difference between the estimated number of deaths that would occur when coverage is expanded to one of the three coverage targets compared to baseline (with no change in coverage) represents the mortality impact (ie, the number of lives saved or stillbirths prevented) attributable to MNCH coverage expansion.

Mortality reduction was examined for mothers, stillbirths, neonates, and children (1–59 months) for 73 countries (see **Online Supplementary Document(Online Supplementary Document)**), and results were combined to quantify regional and global impact. The mortality impact was estimated cumulatively (for the period from 2016–2020) and for the target year (2020). Mortality rates were projected by country, and mortality risk for each sub–group was calculated by applying weights for 2015–2020 according to the number of births projected by the UN Population Division for this time period.

## FINDINGS

If population–level coverage of CHW–provided maternal, neonatal and child health interventions (shown in **Figure 1****)** could expand to reach 50% in 2020 (without reducing the coverage level for those interventions already at a higher level of coverage), an estimated total of 3.0 (sensitivity bounds 1.8–4.0) million lives would be saved during the five–year period from 2016 to 2020. If coverage reached 70% and 90%, the cumulative number of lives saved during this time period would increase to 4.9 (3.1–6.3) and 6.9 (3.7–8.7) million, respectively. Neonates and children 1–59 months of age would be the greatest beneficiaries of increased delivery of interventions at the community level. At the lowest threshold of 50% coverage, one–quarter (27%, 804 470/3 008 900) of the total impact would be among neonates and half (50%, 1 485 650/3 008 900) would be among children aged 1–59 months. A reduction in the number of stillbirths accounts for one–quarter of the impact (23%, 703 470/3 008 900) and the number of maternal lives saved remains relatively small (<1% of the total lives saved). The distribution of relative impact across these MNCH categories was similar for the three levels of coverage expansion which were modeled.

For the target year of 2020 alone, the total number of deaths would be reduced by approximately 32% (compared to the number predicted if population–level coverage of CHW–led interventions remained static) if near–universal coverage (90%) were achieved (**Table 1**). Targeting lower levels of coverage (50% and 70%) would produce 14% and 23% fewer deaths in 2020, respectively. Declines would be greatest among children during the post–neonatal period (1–59 months) with 19%, 29% or 41% fewer deaths in this age group estimated in 2020 if these three targets, respectively, were achieved. If coverage of key interventions reached half (50%) of the neonates and pregnant women, the estimated reduction in mortality for the year 2020 would be 13% fewer neonatal deaths and 12% fewer stillbirths.

**Table 1:** Estimated number of deaths that would be prevented in 2020 if countries expanded the population coverage of evidence–based maternal, neonatal, and child health interventions that community health workers can provide

Type of death	Deaths in 2020 with NO change in intervention coverage (n)	50% coverage		70% coverage		90% coverage	
**Deaths prevented in 2020 (n)**	**% reduction**	**Deaths prevented in 2020 (n)**	**% reduction**	**Deaths prevented in 2020 (n)**	**% reduction**
**Maternal**	297 900	6200	2.1%	12 100	4.1%	19 500	6.5%
**Stillbirth**	2 294 700	280 400	12.2%	420 700	18.3%	565 040	24.6%
**Neonatal**	2 454 000	311 400	12.7%	510 400	20.8%	704 230	28.7%
**Child (1–59 months)**	3 125 100	582 300	18.6%	919 400	29.4%	1 295 160	41.4%
**Total**	**8** **171** **700**	**1** **180** **300**	**14.4%**	**1** **862** **600**	**22.8%**	**2** **583** **930**	**31.6%**
Estimated range*		(697 380–1 541 710)	(8.5%–18.9%)	(1 174 010–2 412 070)	(14.4%–29.5%)	(1 735 790–3 197 900)	(21.2%–39.1%)

*Ranges for impact estimates were produced by conducting sensitivity analyses  that calculated impact of interventions based upon the highest level of effectiveness reported (upper bound) for all interventions compared to the lowest levels of effectiveness reported for all interventions (lower bound).

**Figure 2:**
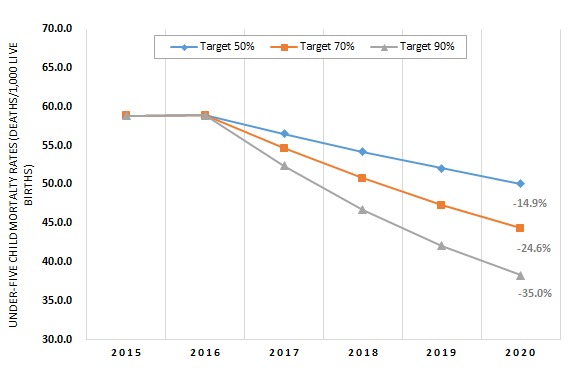
Under–five mortality rate for 73 Countdown countries (weighted by number of births in each country) with intervention scale–up by community health workers to reach population coverage levels of 50%, 70%, or 90%.

**Figure 3:**
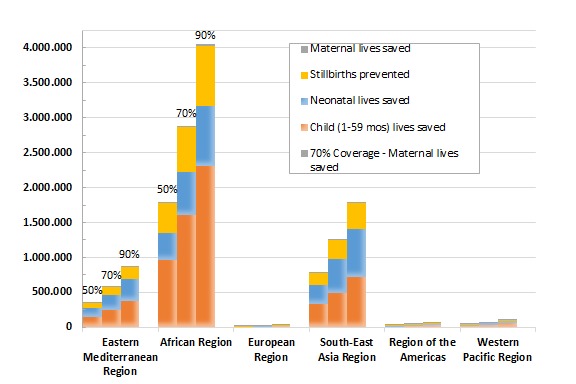
Overall mortality impact (lives saved and stillbirths prevented) by WHO region for 2015–2020 with intervention scale–up by CHWs to reach population coverage of 50%, 70%, and 90%.

The under–five mortality rate (U5MR) in 2020 would drop 15%, 25%, or 35% if coverage in these Countdown countries were increased to 50%, 70%, or 90% respectively. With scale–up to near–universal coverage (90%), the U5MR is projected to decline to 38 deaths per 1000 live births in 2020 compared to 59 deaths per 1000 live births in 2015 for this weighted sample of 73 countries (**Figure 2**). The World Health Organization (WHO) regions with the greatest potential for the number of deaths prevented are the African region and South–East Asia region (**Figure 3**). By expanding coverage to reach just 50% with CHWs in the Countdown countries in just these two regions, 90% of the total maternal deaths preventable globally through the community platform would be prevented, as would 86% of the total stillbirths, 81% of the neonatal deaths, and 88% of the child deaths (between 1–59 months). Individual countries that would benefit the most from scaling up community–based interventions to 90% coverage are India (535 000 lives saved nationally), Nigeria (458 000), Pakistan (162 000), and Democratic Republic of Congo (160 000). The **Online Supplementary Document(Online Supplementary Document)** contains the total numbers of lives saved among mothers, stillbirths, neonates, and children aged 1–59 months by target coverage level.

**Figure 4:**
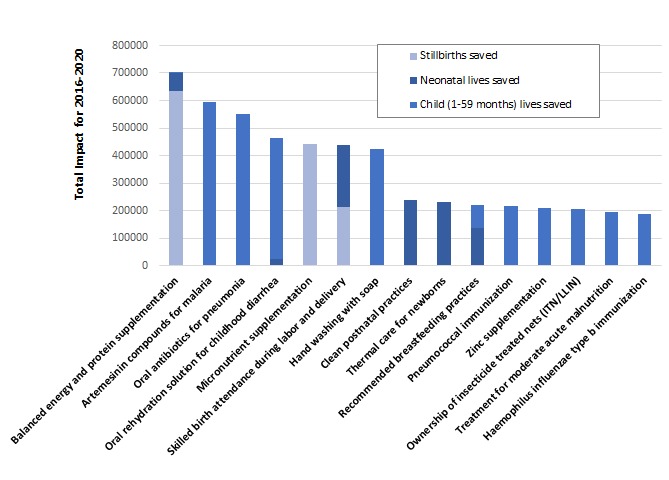
Lives saved and stillbirths prevented by community–based interventions provided by CHWs to reach population coverage of 90% by 2020. The number of maternal lives saved is not shown because the scale of the number is too small to be displayed on the same graph.

At a population coverage level of 90%, the specific interventions included in our analyses contributing to save the greatest number of lives for all sub–groups combined are balanced energy and protein supplementation during pregnancy, artemisinin compounds for treatment of malaria, oral antibiotics for pneumonia, oral rehydration solution (ORS) for diarrhea, and multiple micronutrient supplementation during pregnancy (**Figure 4**). Among the interventions that can be provided by CHWs to reduce the risk of stillbirths, improving nutrition during pregnancy played a central role. Balanced energy and protein supplementation and micronutrient supplementation accounted for the majority (49% and 34%, respectively) of the stillbirths prevented. For newborns, one–quarter of neonatal deaths would be prevented by increasing coverage of clean postnatal care (26%) and another one–quarter by implementing thermal care practices (25%). Among all the deaths among children aged 1–59 months that would be prevented by CHW–led scale–up, 19% would be prevented by anti–malarial treatment, 19% by oral antibiotics for pneumonia, and 14% by ORS. Clean birth practices and calcium supplementation during pregnancy provided by CHWs would account for 92% of the maternal lives saved according to our analysis.

## DISCUSSION

Our findings indicate that over the next decade and beyond, millions of deaths could be averted by expanding the population coverage of specific evidence–based interventions via well–trained and adequately–supported CHWs. A large portion (32%) of the projected 8.2 million deaths in 2020, including fetal, maternal, neonatal, and child (aged 1–59 months) deaths, would be prevented if near–universal (90%) coverage of key interventions is achieved. With more modest improvements in coverage, the achievements would be notable as well: 23% of deaths would be saved at 70% coverage, and 14% would be saved at 50% coverage.

Evidence of the effectiveness of community–based approaches to improve the health of mothers, newborns, and children in geographically–defined populations is abundant [17,18]. Most assessments have been conducted for a limited subset of interventions (rather than a broader package of interventions) which are implemented in small populations over relatively short periods where CHWs are well–trained, supported, and supervised [18]. Ethiopia and Rwanda are arguably the best recent examples showing the benefits for the health of mothers, newborns, and children by deploying CHWs to expand the population coverage of evidence–based interventions [19,20]. Nonetheless, major challenges remain in achieving high levels of coverage of these interventions, even in Ethiopia [21].

Despite the established benefit of known lifesaving interventions, coverage of essential interventions remains surprisingly low in many settings. In Nigeria, with a population of 31 million children younger than 5 years of age, for example, the population coverage of two highly effective interventions remains abysmally low: only 34% of under–5 children with diarrhea are treated with ORS and only 17% of children younger than 6 months of age are exclusively breastfed [22]. Similar findings exist for other critical interventions in many low–income, high–mortality countries.

Relying exclusively on facility–based platforms (primary health centers and hospitals staffed by doctors and nurses) to end preventable child and maternal deaths and to achieve universal coverage of basic and essential services remains a challenge for several reasons. The utilization of facility–based services decreases exponentially as the household’s distance from the location increases, especially if the facility is greater than 3 km or more than 30 minutes away [23]. In many countries, the mean travel time to the nearest facility is more than 30 minutes and in some countries such as Kenya and Ethiopia, considerably more [23]. Construction, staffing, and operating a sufficient number of primary health centers to deliver high–quality care that is readily accessible to the population (within 3 km) and that would be utilized by the majority of the population for the interventions included in our analysis is not achievable in most Countdown countries for the foreseeable future. The logistical and financial challenges are much more daunting than the logistical and financial challenges of building strong CHW programs that complement existing facility–based services.

Existing evidence suggests that using CHWs to deliver essential health services can be a cost–effective approach for health programs in LMICs [24]. Although high–quality community–based programming is not cheap [25], it is the most effective option to reduce mortality (compared to investing solely in facility–based care), as well as the least expensive option we currently have (compared to investing solely in facility–based care) to end preventable child and maternal deaths in resource–limited countries by 2030 [26]. Achieving the same level of coverage of evidence–based interventions by expanding only facility–based services with more numbers of highly trained personnel will take decades longer and cost much more compared to the expansion of community–based health care through CHWs [8,27–30]. CHWs would play an important role as part of any health care system, however, we do not underestimate the challenges of recruiting, training, and retaining enough CHWs to achieve near–universal coverage of evidence–based interventions. This is particularly true for countries that are politically unstable and experiencing conflict. Even in countries with well–developed CHW programs (eg, Ethiopia) coverage of a number of evidence–based interventions remains relatively low [21].

However, in our view, the human resources are available in communities to expand the coverage of community–based MNCH interventions. Experience teaches that people with limited education can learn the skills needed to provide these interventions, and there are adequate numbers of people who are eager and willing to serve their community for the purpose of saving lives. The needed trainers and supervisors can be acquired if the financial investment is adequate.

The organizational challenges of scaling up community–based delivery systems globally in order to reach all households on a regular basis will require innovation and commitment. Identifying the population most at risk and vulnerable subgroup(s) is essential for addressing equity and closing the coverage gap with CHW–led initiatives. Countries such as Brazil, Bangladesh, Ethiopia, Nepal and Rwanda have made major strides to adapt and develop community–based delivery systems with CHW programs, and they have all made major progress in reducing maternal and child mortality [31]. Guidance for addressing the challenges of CHW programming at scale based on these global experiences and others has been compiled and is readily available [32] along with a set of recommendations from an Expert Panel arising from a comprehensive review of the effectiveness of CBPHC programming for improving MNCH [18]. Specifying the details such as how many and what types of CHWs would be needed to achieve and sustain these levels of coverage is beyond the scope of this article.

The strengths of our approach include the use of a validated model that has been used for multi–country assessments [33,34] and draws upon updated country–specific data about mortality and health status to support a global analysis of impact from coverage expansion. Our study has limitations and many relate to the paucity of data available for reliably measuring and tracking change of intervention coverage at a population level. Modelled trends for expanded coverage were drawn as linear increases but more complex patterns due to saturation or synergy effects may emerge and were not examined in our approach. The decision to keep the counterfactual levels of coverage unchanged from baseline levels is a possible methodological weakness of our analysis, but it simplified our work considerably and we had no solid basis for assuming that there would be major secular expansions in coverage of specific interventions barring a major global push of the type we are arguing for in this paper. Furthermore, whatever increases there might be could possibly vary from intervention to intervention and country to country, so accounting for this in our analysis would have been problematic.

Our mortality impact estimates may be somewhat optimistic if effectiveness of certain interventions in LiST is derived from efficacy studies in which interventions are delivered under relatively ideal conditions. On the other hand, our estimates may be considered conservative because we did not estimate or account for the possible benefits of scaling up other community–based interventions, including oral misoprostol for prevention of post–partum hemorrhage, promotion of care seeking for antenatal and delivery care at facilities (highlighted in a recent review [35]), promotion of HIV testing, counseling on prevention of sexually transmitted infections, promotion of immunizations against human papillomavirus and hepatitis B, as well as the promotion and provision of family planning services. The effectiveness of CHWs in scaling up high–quality family planning services – not only oral contraception and condoms but also injectable contraceptives and even subcutaneous implants – has been well–established [36–39] and fewer births can by itself produce a decline in the number of stillbirths and deaths of mothers and neonates by simply decreasing the denominator – the number of pregnant women and newborns. Eliminating the unmet need for family planning alone would reduce maternal deaths globally by 29% simply by reducing the number of pregnancies [40], and increased birth spacing would significantly reduce the risk of death during infancy, particularly among higher–parity mothers [41,42]. The evidence for reducing the number of stillbirths from balanced energy and protein supplementation during pregnancy is not widely known or incorporated into programs, but nonetheless strong [43,44].

Overall, our findings do nonetheless point to the strong benefits of giving priority to strengthening and expanding community–based delivery systems so that the rate of increase in population coverage of evidence–based interventions for mothers, newborns, and children can begin to accelerate. The highest–impact interventions highlighted by our analysis may be used as a starting point to guide prioritization of community–based programming. Recent experience highlights the need to rigorously plan, evaluate, and refine community–based approaches [45].

CHWs can act as not only agents of change who directly provide health care services but also as liaisons who facilitate proper referrals and timely transfer if complications arise to foster an “integrated continuum of care” [7]. There is no “one size fits all” approach to developing, expanding, and strengthening community–based delivery systems. Each country – both the government and civil society – has to fashion approaches that make sense given their human and financial resources, geography, culture, current health system, and epidemiological context. However, learning from successful examples elsewhere can provide the basis for creative thinking and adaptation to fit local circumstances [32,46].

The findings of our study complement those of another recent global MNCH analysis which compared the number of deaths that could be averted by three service delivery platforms: the community platform, the primary health center platform, and the hospital platform [30]. According to this analysis, 21% of the stillbirths, 49% of the neonatal deaths, and 93% of the child deaths that can be averted by delivering currently available evidence–based interventions through a community platform.

Strengthening community–based delivery strategies can provide additional benefits beyond reducing the number of stillbirths and maternal, neonatal and child deaths. Community–based approaches are becoming increasingly important in the prevention, identification, and treatment of HIV infection (and ending the HIV epidemic), tuberculosis, and non–communicable diseases such as hypertension, diabetes, and mental illness as well as medical care of the elderly [8,47,48]. Furthermore, robust CHW programs have the potential to support identification of infectious disease outbreaks early on, with important savings not only in lives but in prevention of economic meltdowns [49].

For decades, CHWs have been seen by many as a second–class temporary solution for second–class citizens and therefore not viewed as viable long–term members of the national health workforce, especially once countries pass through the epidemiological transition and maternal and child health are no longer epidemiological priorities. However, with not only the growing evidence of the effectiveness of CHWs for improving maternal and child health services but also the increasing recognition of the CHW potential for addressing the growing burden of chronic, non–communicable diseases in developing countries [50], as well as in high–income settings such as the United States to address persistent health disparities [8,51], the tide is now shifting toward the view that CHWs are a long–term asset and essential for high–performing health systems everywhere [10].

## CONCLUSIONS

The full potential of community–based approaches to improve population health through CHWs is only beginning to be appreciated by the global health community. Better utilization of existing CHWs, establishing new CHW programs where none are now present, expanding the CHW workforce, creating attractive long–term career development opportunities for CHWs, and strengthening the overall quality of CHW programs will all be required to achieve the full potential of community–based programming for mortality reduction. The three specific interventions that could save the most lives by expanding coverage to 90% are balanced energy and protein supplementation for pregnant women, antibiotic treatment of childhood malaria, and childhood treatment of pneumonia. These are high–impact interventions partly because the current levels of coverage are so low (in contrast to, for instance, immunizations, where current levels of coverage are quite high).

If near–universal (90%) coverage of evidence–based interventions for mothers and children were achieved in 73 Countdown countries, 6.9 million lives would be saved during the period from 2016 to 2020, and the overall number of death would be reduced by 41% compared to baseline levels. The full cost of achieving this goal would be far less than the cost of reaching this level of coverage through expansion of facility–based care provided by higher–level providers.
