# Supplementary material for: Expanding the population coverage of evidence–based interventions with community health workers to save the lives of mothers and children: an analysis of potential global impact using the Lives Saved Tool (LiST)
Source: J Glob Health. 2017 Sep 15;7(2):020401. doi: 10.7189/jogh.07.020401 (PMC5592116; doi:10.7189/jogh.07.020401)
Supplement: Online Supplementary Document [file jogh-07-020401-s001.pdf]

Online Supplementary Document

Chou et al. Expanding the population coverage of evidence–based interventions with community health workers to save the lives of mothers and children: an analysis of potential global impact using the Lives Saved Tool (LiST)

J Glob Health 2017;7:020401

APPENDIX

Country, regional, and global estimates of maternal deaths, stillbirths, child (neonatal and 1-59-month-old) deaths that would be prevented by expanded coverage of community-based interventions provided by community health workers

This appendix contains country, regional, and global estimates of the number of lives that could be saved by the implementation of evidence-based interventions by community-level providers outside of health facilities. Table 1 lists all the countries that were included in the analysis. Table 2 lists the interventions included in the analysis. Tables 3-7 provide estimates for each category of deaths (maternal deaths, stillbirths, neonatal deaths, and deaths of children aged 1-59-months). The estimated number of deaths for each population subgroup and the number of projected lives saved in 2020 given a level of *x* coverage (50%, 70%, and 90%) is provided and the mortality impact is presented as a calculated percentage. There are minor differences in these tables compared to the main pages due to rounding.

The column header is labeled as the "minimum" for population coverage because in countries where the current level of coverage for an intervention exceeds the designated target, coverage was <u>not</u> modeled to decline and the existing coverage level was entered into the model. Table 8 reports the total impact for all population subgroups combined.

Contents

Table 1. Low-income high-burden countries (N=73) included in analysis 3

Table 2. Interventions that can be provided by community health workers with mortality impact estimated using the Lives Saved Tool (LiST) 4

Table 3. Estimates of numbers and percentages of maternal deaths that would be saved by expanded coverage of community-based interventions, by country and WHO region 6

Table 4. Estimates of numbers and percentages of stillbirths that would be averted by expanded coverage of community-based interventions, by country and WHO region 10

Table 5. Estimates of numbers and percentages of neonatal deaths that would be saved by expanded coverage of community-based interventions, by country and WHO region 14

Table 6. Estimates of numbers and percentages of child (1-59 months) deaths that would be saved by expanded coverage of community-based interventions, by country and WHO region 18

Table 7. Estimates of numbers and percentages of deaths of all under-five children (0-59 months) that would be saved by expanded coverage of community-based interventions, by country and WHO region 22

Table 8. Estimates of numbers and percentages of ALL deaths (maternal, child and stillbirths combined) that would be averted by expanded coverage of community-based interventions, by country and WHO region 26

Table 1. Low-income high-burden countries (N=73) included in analysis

COUNTRIES BY WHO REGIONS
**African Region**
Angola
Benin
Botswana
Burkina Faso
Burundi
Cameroon
Central African Republic
Chad
Comoros
Congo
Côte d'Ivoire
Democratic Republic of the Congo
Equatorial Guinea
Eritrea
Ethiopia
Gabon
Gambia
Ghana
Guinea
Guinea-Bissau
Kenya
Lesotho
Liberia
Madagascar
Malawi
Mali
Mauritania
Mozambique
Niger
Nigeria
Rwanda
São Tomé and Príncipe
Senegal
Sierra Leone
South Africa
South Sudan
Swaziland
Togo
Uganda
United Republic of Tanzania
Zambia
Zimbabwe
**Region of the Americas**
Bolivia
Brazil
Guatemala
Haiti
Peru
**South-East Asia Region**
Bangladesh
Dem. People's Republic of Korea
India
Indonesia
Myanmar
Nepal
**European Region**
Azerbaijan
Kyrgyzstan
Tajikistan
Turkmenistan
Uzbekistan
**Eastern Mediterranean Region**
Afghanistan
Djibouti
Egypt
Iraq
Morocco
Pakistan
Somalia
Sudan
Yemen
**Western Pacific Region**
Cambodia
Lao People's Democratic Republic
Papua New Guinea
Philippines
Solomon Islands
Viet Nam

Table 2. Interventions that can be provided by community health workers with mortality impact estimated using the Lives Saved Tool (LiST)

	Intervention	Intervention Impact			
		Maternal	Stillbirths	Neonates	Children (1-59 m)
PREPREGNANCY	Folic acid via supplementation or fortification			✓	
PREGNANCY	Tetanus toxoid immunization	✓		✓	
	Intermittent preventive treatment in pregnancy (IPTp)	✓	✓	✓	✓
	Calcium supplementation	✓			
	Syphilis detection and treatment		✓	✓	
	Micronutrient supplementation		✓	✓	✓
	Balanced energy and protein supplementation		✓	✓	✓
BIRTH	Skilled birth attendance	✓	✓	✓	
	Clean birth practices	✓		✓	
	Immediate assessment and stimulation			✓	
	Neonatal resuscitation			✓	
NEONATAL	Promotion of breastfeeding			✓	✓
	Clean postnatal practices			✓	

	Chlorhexidine umbilical cord treatment			✓	
	Thermal care			✓	
	Oral antibiotics for neonatal sepsis			✓	
CHILDHOOD - PREVENTION	Education and provision of complementary foods			✓	✓
	Vitamin A supplementation			✓	✓
	Zinc supplementation			✓	✓
	Immunization for vaccine-preventable diseases			✓	✓
	Hand washing with soap			✓	✓
	Safe disposal of children's stools			✓	✓
	Use of insecticide-treated nets or indoor residual spraying			✓	✓
CHILDHOOD - TREATMENT	Oral rehydration solution for childhood diarrhea			✓	✓
	Zinc for treatment of diarrhea			✓	✓
	Antibiotics for dysentery			✓	✓
	Oral antibiotics for pneumonia			✓	✓
	Treatment for moderate acute malnutrition			✓	✓
	Vitamin A for treatment of measles			✓	✓
	Artemisinin compounds for malaria treatment			✓	✓

Table 3. Estimates of numbers and percentages of maternal deaths that would be saved by expanded coverage of community-based interventions, by country and WHO region

	Number of maternal deaths at baseline and at various levels of population coverage in 2020					Number of maternal deaths prevented at various levels of population coverage				Percentage of maternal deaths prevented at various levels of population coverage		
**Region/Country**	**No change**	**Target 50%**	**Target 70%**	**Target 90%**		**Target 50%**	**Target 70%**	**Target 90%**		**Target 50%**	**Target 70%**	**Target 90%**
**African Region**	**203,614**	**198,670**	**194,695**	**189,585**		**4,944**	**8,919**	**14,029**		**2.4%**	**4.4%**	**6.9%**
Angola	5,903	5,782	5,625	5,463		121	278	440		2.0%	4.7%	7.5%
Benin	1,625	1,599	1,588	1,543		26	37	82		1.6%	2.3%	5.0%
Botswana	66	66	65	65		0	1	1		0.0%	1.5%	1.5%
Burkina Faso	2,748	2,692	2,669	2,591		56	79	157		2.0%	2.9%	5.7%
Burundi	3,716	3,653	3,610	3,507		63	106	209		1.7%	2.9%	5.6%
Cameroon	5,248	5,156	5,078	4,897		92	170	351		1.8%	3.2%	6.7%
Central African Republic	1,470	1,443	1,408	1,367		27	62	103		1.8%	4.2%	7.0%
Chad	5,778	5,547	5,409	5,269		231	369	509		4.0%	6.4%	8.8%
Comoros	90	89	88	87		1	2	3		1.1%	2.2%	3.3%
Congo	762	747	741	734		15	21	28		2.0%	2.8%	3.7%
Côte d'Ivoire	5,507	5,398	5,302	5,145		109	205	362		2.0%	3.7%	6.6%
Democratic Republic of the Congo	23,613	23,134	22,929	22,448		479	684	1,165		2.0%	2.9%	4.9%
Equatorial Guinea	111	109	108	105		2	3	6		1.8%	2.7%	5.4%
Eritrea	880	854	834	814		26	46	66		3.0%	5.2%	7.5%
Ethiopia	11,550	11,053	10,817	10,574		497	733	976		4.3%	6.3%	8.5%
Gabon	152	148	147	145		4	5	7		2.6%	3.3%	4.6%
Gambia	622	611	599	581		11	23	41		1.8%	3.7%	6.6%
Ghana	2,763	2,728	2,714	2,648		35	49	115		1.3%	1.8%	4.2%
Guinea	3,223	3,148	3,063	2,977		75	160	246		2.3%	5.0%	7.6%

Region/Country	Number of maternal deaths at baseline and at various levels of population coverage in 2020					Number of maternal deaths prevented at various levels of population coverage				Percentage of maternal deaths prevented at various levels of population coverage		
	No change	Target 50%	Target 70%	Target 90%		Target 50%	Target 70%	Target 90%		Target 50%	Target 70%	Target 90%
Guinea-Bissau	385	375	365	355		10	20	30		2.6%	5.2%	7.8%
Kenya	8,284	8,120	7,984	7,748		164	300	536		2.0%	3.6%	6.5%
Lesotho	298	294	290	282		4	8	16		1.3%	2.7%	5.4%
Liberia	1,188	1,168	1,150	1,114		20	38	74		1.7%	3.2%	6.2%
Madagascar	3,162	3,081	3,000	2,916		81	162	246		2.6%	5.1%	7.8%
Malawi	4,832	4,756	4,715	4,660		76	117	172		1.6%	2.4%	3.6%
Mali	4,686	4,593	4,510	4,373		93	176	313		2.0%	3.8%	6.7%
Mauritania	845	831	821	797		14	24	48		1.7%	2.8%	5.7%
Mozambique	5,835	5,742	5,615	5,446		93	220	389		1.6%	3.8%	6.7%
Niger	6,275	6,071	5,915	5,756		204	360	519		3.3%	5.7%	8.3%
Nigeria	60,331	58,595	57,048	55,495		1,736	3,283	4,836		2.9%	5.4%	8.0%
Rwanda	1,036	1,015	1,006	982		21	30	54		2.0%	2.9%	5.2%
São Tomé and Príncipe	11	11	11	11		0	0	0		0.0%	0.0%	0.0%
Senegal	1,854	1,821	1,785	1,732		33	69	122		1.8%	3.7%	6.6%
Sierra Leone	3,126	3,071	3,018	2,925		55	108	201		1.8%	3.5%	6.4%
South Africa	1,465	1,444	1,435	1,425		21	30	40		1.4%	2.0%	2.7%
South Sudan	3,762	3,656	3,600	3,542		106	162	220		2.8%	4.3%	5.8%
Swaziland	132	130	129	128		2	3	4		1.5%	2.3%	3.0%
Togo	943	927	910	882		16	33	61		1.7%	3.5%	6.5%
Uganda	6,451	6,328	6,208	6,028		123	243	423		1.9%	3.8%	6.6%
United Republic of Tanzania	8,902	8,766	8,505	8,239		136	397	663		1.5%	4.5%	7.4%
Zambia	1,631	1,606	1,587	1,539		25	44	92		1.5%	2.7%	5.6%
Zimbabwe	2,353	2,312	2,294	2,250		41	59	103		1.7%	2.5%	4.4%

	Number of maternal deaths at baseline and at various levels of population coverage in 2020					Number of maternal deaths prevented at various levels of population coverage				Percentage of maternal deaths prevented at various levels of population coverage		
**Region/Country**	**No change**	**Target 50%**	**Target 70%**	**Target 90%**		**Target 50%**	**Target 70%**	**Target 90%**		**Target 50%**	**Target 70%**	**Target 90%**
**Region of the Americas**	**3,476**	**3,397**	**3,346**	**3,286**		**79**	**130**	**190**		**2.3%**	**3.7%**	**5.5%**
Bolivia	522	515	512	501		7	10	21		1.3%	1.9%	4.0%
Brazil	1,238	1,210	1,199	1,188		28	39	50		2.3%	3.2%	4.0%
Guatemala	385	377	368	358		8	17	27		2.1%	4.4%	7.0%
Haiti	932	903	878	852		29	54	80		3.1%	5.8%	8.6%
Peru	399	392	389	387		7	10	12		1.8%	2.5%	3.0%
**South-East Asia Region**	**58,518**	**57,908**	**56,506**	**54,894**		**610**	**2,012**	**3,624**		**1.0%**	**3.4%**	**6.2%**
Bangladesh	5,283	5,201	5,155	5,109		82	128	174		1.6%	2.4%	3.3%
Dem. People's Republic of Korea	300	296	294	292		4	6	8		1.3%	2.0%	2.7%
India	43,787	43,390	42,098	40,645		397	1,689	3,142		0.9%	3.9%	7.2%
Indonesia	5,979	5,896	5,863	5,810		83	116	169		1.4%	1.9%	2.8%
Myanmar	1,679	1,658	1,650	1,617		21	29	62		1.3%	1.7%	3.7%
Nepal	1,490	1,467	1,446	1,421		23	44	69		1.5%	3.0%	4.6%
**European Region**	**482**	**473**	**469**	**464**		**9**	**13**	**18**		**1.9%**	**2.7%**	**3.7%**
Azerbaijan	39	38	38	38		1	1	1		2.6%	2.6%	2.6%
Kyrgyzstan	105	102	101	100		3	4	5		2.9%	3.8%	4.8%
Tajikistan	81	80	79	78		1	2	3		1.2%	2.5%	3.7%
Turkmenistan	42	42	42	41		0	0	1		0.0%	0.0%	2.4%
Uzbekistan	215	211	209	207		4	6	8		1.9%	2.8%	3.7%
**Eastern Mediterranean Region**	**26,958**	**26,498**	**26,074**	**25,528**		**460**	**884**	**1,430**		**1.7%**	**3.3%**	**5.3%**

	Number of maternal deaths at baseline and at various levels of population coverage in 2020					Number of maternal deaths prevented at various levels of population coverage				Percentage of maternal deaths prevented at various levels of population coverage		
**Region/Country**	**No change**	**Target 50%**	**Target 70%**	**Target 90%**		**Target 50%**	**Target 70%**	**Target 90%**		**Target 50%**	**Target 70%**	**Target 90%**
Afghanistan	4,198	4,107	4,048	3,987		91	150	211		2.2%	3.6%	5.0%
Djibouti	47	46	45	45		1	2	2		2.1%	4.3%	4.3%
Egypt	767	752	746	740		15	21	27		2.0%	2.7%	3.5%
Iraq	655	648	645	642		7	10	13		1.1%	1.5%	2.0%
Morocco	777	765	760	744		12	17	33		1.5%	2.2%	4.2%
Pakistan	9,463	9,375	9,187	8,924		88	276	539		0.9%	2.9%	5.7%
Somalia	3,736	3,607	3,516	3,423		129	220	313		3.5%	5.9%	8.4%
Sudan	4,043	3,982	3,958	3,903		61	85	140		1.5%	2.1%	3.5%
Yemen	3,272	3,216	3,169	3,120		56	103	152		1.7%	3.1%	4.6%
**Western Pacific Region**	**4,846**	**4,760**	**4,721**	**4,662**		**86**	**125**	**184**		**1.8%**	**2.6%**	**3.8%**
Cambodia	577	569	566	563		8	11	14		1.4%	1.9%	2.4%
Lao People's Democratic Republic	343	337	332	328		6	11	15		1.7%	3.2%	4.4%
Papua New Guinea	481	474	468	461		7	13	20		1.5%	2.7%	4.2%
Philippines	2,625	2,571	2,550	2,510		54	75	115		2.1%	2.9%	4.4%
Solomon Islands	19	19	19	19		0	0	0		0.0%	0.0%	0.0%
Viet Nam	801	790	786	781		11	15	20		1.4%	1.9%	2.5%
**Grand Total**	**297,894**	**291,706**	**285,811**	**278,419**		**6,188**	**12,083**	**19,475**		**2.1%**	**4.1%**	**6.5%**

Table 4. Estimates of numbers and percentages of stillbirths that would be averted by expanded coverage of community-based interventions, by country and WHO region

	Number of stillbirths at baseline and at various levels of population coverage in 2020					Number of stillbirths prevented at various levels of population coverage				Percentage of stillbirths prevented at various levels of population coverage		
**Region/Country**	**No change**	**Target 50**	**Target 70**	**Target 90**		**Target 50**	**Target 70**	**Target 90**		**Target 50**	**Target 70**	**Target 90**
**African Region**	**1,043,170**	**868,840**	**787,850**	**705,490**		**174,330**	**255,320**	**337,680**		**16.7%**	**24.5%**	**32.4%**
Angola	33,880	30,950	28,900	26,920		2,930	4,980	6,960		8.6%	14.7%	20.5%
Benin	12,060	10,010	9,150	8,140		2,050	2,910	3,920		17.0%	24.1%	32.5%
Botswana	780	710	690	660		70	90	120		9.0%	11.6%	15.5%
Burkina Faso	15,970	13,010	11,880	10,470		2,960	4,090	5,500		18.5%	25.6%	34.4%
Burundi	14,000	11,250	10,120	8,840		2,750	3,880	5,160		19.6%	27.7%	36.9%
Cameroon	16,920	14,780	13,690	12,340		2,140	3,230	4,580		12.6%	19.1%	27.1%
Central African Republic	5,670	4,580	4,000	3,440		1,090	1,670	2,230		19.2%	29.5%	39.3%
Chad	26,910	22,380	20,340	18,420		4,530	6,570	8,490		16.8%	24.4%	31.5%
Comoros	810	750	730	700		60	80	110		7.4%	9.8%	13.5%
Congo	2,610	2,290	2,150	2,000		320	460	610		12.3%	17.6%	23.4%
Côte d'Ivoire	22,490	19,540	18,030	16,300		2,950	4,460	6,190		13.1%	19.8%	27.5%
Democratic Republic of the Congo	92,490	72,140	64,270	55,850		20,350	28,220	36,640		22.0%	30.5%	39.6%
Equatorial Guinea	520	500	490	460		20	30	60		3.8%	5.7%	11.5%
Eritrea	3,900	3,310	3,030	2,770		590	870	1,130		15.1%	22.3%	29.0%
Ethiopia	96,310	82,100	76,420	71,010		14,210	19,890	25,300		14.8%	20.7%	26.3%
Gabon	720	640	600	570		80	120	150		11.2%	16.8%	20.9%
Gambia	2,130	1,850	1,690	1,500		280	440	630		13.1%	20.7%	29.6%
Ghana	19,460	17,650	16,870	15,120		1,810	2,590	4,340		9.3%	13.3%	22.3%
Guinea	10,060	8,570	7,750	6,990		1,490	2,310	3,070		14.8%	23.0%	30.5%

Region/Country	Number of stillbirths at baseline and at various levels of population coverage in 2020					Number of stillbirths prevented at various levels of population coverage				Percentage of stillbirths prevented at various levels of population coverage		
	**No change**	**Target 50**	**Target 70**	**Target 90**		**Target 50**	**Target 70**	**Target 90**		**Target 50**	**Target 70**	**Target 90**
Guinea-Bissau	2,480	1,970	1,730	1,510		510	750	970		20.5%	30.2%	39.1%
Kenya	36,410	31,510	29,090	26,280		4,900	7,320	10,130		13.5%	20.1%	27.8%
Lesotho	1,190	1,000	910	820		190	280	370		16.0%	23.6%	31.2%
Liberia	3,430	2,820	2,500	2,150		610	930	1,280		17.8%	27.1%	37.3%
Madagascar	16,040	12,250	10,540	8,970		3,790	5,500	7,070		23.6%	34.3%	44.1%
Malawi	16,390	13,400	12,030	10,560		2,990	4,360	5,830		18.2%	26.6%	35.6%
Mali	25,990	21,680	19,650	17,420		4,310	6,340	8,570		16.6%	24.4%	33.0%
Mauritania	3,740	3,490	3,360	3,160		250	380	580		6.7%	10.2%	15.5%
Mozambique	22,530	17,910	15,730	13,580		4,620	6,800	8,950		20.5%	30.2%	39.7%
Niger	41,980	34,800	31,270	27,980		7,180	10,710	14,000		17.1%	25.5%	33.4%
Nigeria	312,850	254,990	227,810	202,530		57,860	85,040	110,320		18.5%	27.2%	35.3%
Rwanda	6,180	5,060	4,620	4,110		1,120	1,560	2,070		18.1%	25.2%	33.5%
São Tomé and Príncipe	120	110	100	90		10	20	30		8.5%	16.9%	25.4%
Senegal	14,260	12,420	11,320	10,130		1,840	2,940	4,130		12.9%	20.6%	29.0%
Sierra Leone	5,510	4,680	4,200	3,680		830	1,310	1,830		15.1%	23.8%	33.2%
South Africa	18,480	17,060	16,510	15,950		1,420	1,970	2,530		7.7%	10.7%	13.7%
South Sudan	14,130	11,470	10,330	9,270		2,660	3,800	4,860		18.8%	26.9%	34.4%
Swaziland	410	360	340	320		50	70	90		12.1%	16.9%	21.7%
Togo	8,780	7,410	6,650	5,840		1,370	2,130	2,940		15.6%	24.3%	33.5%
Uganda	38,790	33,680	30,940	27,910		5,110	7,850	10,880		13.2%	20.2%	28.1%
United Republic of Tanzania	49,680	41,990	37,390	33,100		7,690	12,290	16,580		15.5%	24.7%	33.4%
Zambia	15,250	12,660	11,560	9,940		2,590	3,690	5,310		17.0%	24.2%	34.8%
Zimbabwe	10,880	9,160	8,490	7,690		1,720	2,390	3,190		15.8%	22.0%	29.3%
**Region of the Americas**	**44,180**	**41,090**	**39,820**	**38,520**		**3,090**	**4,360**	**5,660**		**7.0%**	**9.9%**	**12.8%**

	Number of stillbirths at baseline and at various levels of population coverage in 2020					Number of stillbirths prevented at various levels of population coverage				Percentage of stillbirths prevented at various levels of population coverage		
**Region/Country**	**No change**	**Target 50**	**Target 70**	**Target 90**		**Target 50**	**Target 70**	**Target 90**		**Target 50**	**Target 70**	**Target 90**
Bolivia	3,240	3,040	2,960	2,860		200	280	380		6.2%	8.7%	11.7%
Brazil	24,200	22,890	22,370	21,840		1,310	1,830	2,360		5.4%	7.6%	9.8%
Guatemala	5,190	4,840	4,670	4,500		350	520	690		6.7%	10.0%	13.3%
Haiti	6,310	5,340	4,940	4,560		970	1,370	1,750		15.4%	21.7%	27.8%
Peru	5,250	4,980	4,870	4,760		270	380	490		5.1%	7.2%	9.3%
**South-East Asia Region**	**745,880**	**678,690**	**639,160**	**599,090**		**67,190**	**106,720**	**146,790**		**9.0%**	**14.3%**	**19.7%**
Bangladesh	73,770	63,740	58,610	53,740		10,030	15,160	20,030		13.6%	20.5%	27.2%
Dem. People's Republic of Korea	4,740	4,100	3,860	3,620		640	880	1,120		13.5%	18.6%	23.6%
India	576,150	526,600	495,380	463,910		49,550	80,770	112,240		8.6%	14.0%	19.5%
Indonesia	62,290	57,600	55,770	53,670		4,690	6,520	8,620		7.5%	10.5%	13.8%
Myanmar	18,630	17,120	16,520	15,710		1,510	2,110	2,920		8.1%	11.3%	15.7%
Nepal	10,300	9,540	9,030	8,460		760	1,270	1,840		7.4%	12.3%	17.9%
**European Region**	**16,510**	**14,650**	**13,930**	**13,220**		**1,860**	**2,580**	**3,290**		**11.3%**	**15.6%**	**19.9%**
Azerbaijan	2,530	2,400	2,350	2,300		130	180	230		5.1%	7.1%	9.1%
Kyrgyzstan	1,460	1,390	1,360	1,330		70	100	130		4.8%	6.8%	8.9%
Tajikistan	3,510	3,320	3,240	3,160		190	270	350		5.4%	7.7%	10.0%
Turkmenistan	1,730	1,510	1,430	1,350		220	300	380		12.7%	17.3%	21.9%
Uzbekistan	7,280	6,020	5,550	5,080		1,260	1,730	2,200		17.3%	23.8%	30.2%
**Eastern Mediterranean Region**	**393,060**	**362,840**	**346,550**	**328,500**		**30,220**	**46,510**	**64,560**		**7.7%**	**11.8%**	**16.4%**
Afghanistan	27,970	23,690	21,710	19,830		4,280	6,260	8,140		15.3%	22.4%	29.1%
Djibouti	710	650	630	610		60	80	100		8.4%	11.2%	14.0%
Egypt	28,270	25,990	25,100	24,220		2,280	3,170	4,050		8.1%	11.2%	14.3%

	Number of stillbirths at baseline and at various levels of population coverage in 2020				Number of stillbirths prevented at various levels of population coverage			Percentage of stillbirths prevented at various levels of population coverage		
**Region/Country**	**No change**	**Target 50**	**Target 70**	**Target 90**	**Target 50**	**Target 70**	**Target 90**	**Target 50**	**Target 70**	**Target 90**
Iraq	20,260	19,130	18,670	18,220	1,130	1,590	2,040	5.6%	7.8%	10.1%
Morocco	15,580	14,780	14,460	13,990	800	1,120	1,590	5.1%	7.2%	10.2%
Pakistan	226,090	212,230	202,940	192,180	13,860	23,150	33,910	6.1%	10.2%	15.0%
Somalia	17,970	14,560	13,110	11,760	3,410	4,860	6,210	19.0%	27.0%	34.6%
Sudan	31,650	29,310	28,390	27,080	2,340	3,260	4,570	7.4%	10.3%	14.4%
Yemen	24,560	22,500	21,540	20,620	2,060	3,020	3,940	8.4%	12.3%	16.0%
**Western Pacific Region**	**51,900**	**48,220**	**46,680**	**44,840**	**3,680**	**5,220**	**7,060**	**7.1%**	**10.1%**	**13.6%**
Cambodia	4,270	4,020	3,930	3,820	250	340	450	5.9%	8.0%	10.6%
Lao People's Democratic Republic	4,080	3,640	3,430	3,230	440	650	850	10.8%	15.9%	20.8%
Papua New Guinea	3,530	3,090	2,870	2,660	440	660	870	12.5%	18.7%	24.7%
Philippines	25,030	23,280	22,590	21,600	1,750	2,440	3,430	7.0%	9.7%	13.7%
Solomon Islands	290	240	220	200	50	70	90	17.0%	23.8%	30.6%
Viet Nam	14,710	13,950	13,640	13,330	760	1,070	1,380	5.2%	7.3%	9.4%
Grand Total	2,294,710	2,014,320	1,873,980	1,729,670	280,390	420,730	565,040	12.2%	18.3%	24.6%

Table 5. Estimates of numbers and percentages of neonatal deaths that would be saved by expanded coverage of community-based interventions, by country and WHO region

	Number of neonatal deaths at baseline and at various levels of population coverage in 2020					Number of neonatal deaths prevented at various levels of population coverage				Percentage of neonatal deaths prevented at various levels of population coverage		
Region/Country	No change	Target 50	Target 70	Target 90		Target 50	Target 70	Target 90		Target 50	Target 70	Target 90
**African Region**	**1,034,940**	**889,400**	**806,960**	**720,610**		**145,540**	**227,980**	**314,330**		**14.1%**	**22.0%**	**30.4%**
Angola	60,450	52,880	46,560	40,980		7,570	13,890	19,470		12.5%	23.0%	32.2%
Benin	12,660	11,440	10,960	9,910		1,220	1,700	2,750		9.6%	13.4%	21.7%
Botswana	1,120	1,060	1,030	1,010		60	90	110		5.4%	8.1%	9.8%
Burkina Faso	20,120	17,530	16,730	14,950		2,590	3,390	5,170		12.9%	16.9%	25.7%
Burundi	15,060	13,320	12,650	11,340		1,740	2,410	3,720		11.6%	16.0%	24.7%
Cameroon	22,180	20,030	19,030	16,830		2,150	3,150	5,350		9.7%	14.2%	24.1%
Central African Republic	7,020	6,070	5,390	4,720		950	1,630	2,300		13.5%	23.2%	32.8%
Chad	26,520	20,930	18,190	15,790		5,590	8,330	10,730		21.1%	31.4%	40.5%
Comoros	910	820	790	750		90	120	160		9.9%	13.2%	17.6%
Congo	3,110	2,920	2,840	2,750		190	270	360		6.1%	8.7%	11.6%
Côte d'Ivoire	31,930	28,080	25,910	23,000		3,850	6,020	8,930		12.1%	18.9%	28.0%
Democratic Republic of the Congo	101,980	92,000	88,660	82,200		9,980	13,320	19,780		9.8%	13.1%	19.4%
Equatorial Guinea	1,070	970	940	850		100	130	220		9.3%	12.1%	20.6%
Eritrea	3,190	2,660	2,360	2,130		530	830	1,060		16.6%	26.0%	33.2%
Ethiopia	89,810	69,800	61,220	53,530		20,010	28,590	36,280		22.3%	31.8%	40.4%
Gabon	1,190	1,100	1,060	1,020		90	130	170		7.6%	11.0%	14.3%
Gambia	2,670	2,330	2,130	1,900		340	540	770		12.8%	20.3%	28.9%
Ghana	24,260	22,100	21,350	19,150		2,160	2,910	5,110		8.9%	12.0%	21.1%
Guinea	14,930	12,780	11,180	9,880		2,150	3,750	5,050		14.4%	25.1%	33.8%

	Number of neonatal deaths at baseline and at various levels of population coverage in 2020				Number of neonatal deaths prevented at various levels of population coverage			Percentage of neonatal deaths prevented at various levels of population coverage		
**Region/Country**	**No change**	**Target 50**	**Target 70**	**Target 90**	**Target 50**	**Target 70**	**Target 90**	**Target 50**	**Target 70**	**Target 90**
Guinea-Bissau	2,690	2,300	2,010	1,750	390	680	940	14.5%	25.3%	35.0%
Kenya	35,950	32,450	30,260	27,000	3,500	5,690	8,950	9.7%	15.8%	24.9%
Lesotho	1,990	1,770	1,650	1,470	220	340	520	11.1%	17.1%	26.2%
Liberia	3,860	3,430	3,170	2,790	430	690	1,070	11.1%	17.9%	27.7%
Madagascar	17,370	14,780	12,980	11,570	2,590	4,390	5,800	14.9%	25.3%	33.4%
Malawi	16,400	15,440	15,080	14,420	960	1,320	1,980	5.9%	8.0%	12.1%
Mali	30,230	25,760	23,500	20,630	4,470	6,730	9,600	14.8%	22.3%	31.8%
Mauritania	4,930	4,260	4,040	3,620	670	890	1,310	13.6%	18.1%	26.6%
Mozambique	31,970	27,120	24,360	21,530	4,850	7,610	10,440	15.2%	23.8%	32.7%
Niger	30,660	25,020	21,750	19,180	5,640	8,910	11,480	18.4%	29.1%	37.4%
Nigeria	250,170	210,480	183,280	161,980	39,690	66,890	88,190	15.9%	26.7%	35.3%
Rwanda	6,680	5,960	5,770	5,330	720	910	1,350	10.8%	13.6%	20.2%
São Tomé and Príncipe	120	120	120	110	0	0	10	0.0%	0.0%	8.1%
Senegal	12,110	10,830	9,940	8,840	1,280	2,170	3,270	10.6%	17.9%	27.0%
Sierra Leone	7,880	6,930	6,360	5,580	950	1,520	2,300	12.1%	19.3%	29.2%
South Africa	11,700	10,900	10,610	10,320	800	1,090	1,380	6.8%	9.3%	11.8%
South Sudan	18,450	14,590	12,620	10,770	3,860	5,830	7,680	20.9%	31.6%	41.6%
Swaziland	480	450	440	420	30	40	60	6.3%	8.4%	12.6%
Togo	6,860	6,160	5,670	4,980	700	1,190	1,880	10.2%	17.4%	27.4%
Uganda	34,550	30,540	28,290	25,180	4,010	6,260	9,370	11.6%	18.1%	27.1%
United Republic of Tanzania	41,710	36,150	32,130	28,750	5,560	9,580	12,960	13.3%	23.0%	31.1%
Zambia	15,620	13,910	13,140	11,690	1,710	2,480	3,930	10.9%	15.9%	25.2%
Zimbabwe	12,420	11,260	10,850	10,030	1,160	1,570	2,390	9.3%	12.6%	19.2%
**Region of the Americas**	**47,040**	**43,150**	**41,260**	**39,260**	**3,890**	**5,780**	**7,780**	**8.3%**	**12.3%**	**16.5%**

Region/Country	Number of neonatal deaths at baseline and at various levels of population coverage in 2020					Number of neonatal deaths prevented at various levels of population coverage				Percentage of neonatal deaths prevented at various levels of population coverage		
	No change	Target 50	Target 70	Target 90		Target 50	Target 70	Target 90		Target 50	Target 70	Target 90
Bolivia	4,920	4,580	4,500	4,130		340	420	790		6.9%	8.5%	16.1%
Brazil	25,060	23,410	22,770	22,180		1,650	2,290	2,880		6.6%	9.1%	11.5%
Guatemala	5,840	5,170	4,750	4,340		670	1,090	1,500		11.5%	18.7%	25.7%
Haiti	6,430	5,400	4,720	4,190		1,030	1,710	2,240		16.0%	26.6%	34.8%
Peru	4,780	4,600	4,530	4,430		180	250	350		3.8%	5.2%	7.3%
**South-East Asia Region**	**867,140**	**760,250**	**682,830**	**616,340**		**106,890**	**184,310**	**250,800**		**12.3%**	**21.3%**	**28.9%**
Bangladesh	67,670	58,100	50,910	45,160		9,570	16,760	22,510		14.1%	24.8%	33.3%
Dem. People's Republic of Korea	4,740	4,360	4,220	4,090		380	520	650		8.0%	11.0%	13.7%
India	694,000	606,760	540,850	486,220		87,240	153,150	207,780		12.6%	22.1%	29.9%
Indonesia	63,720	58,220	56,120	53,250		5,500	7,600	10,470		8.6%	11.9%	16.4%
Myanmar	24,590	21,730	20,650	18,680		2,860	3,940	5,910		11.6%	16.0%	24.0%
Nepal	12,420	11,080	10,070	8,940		1,340	2,350	3,480		10.8%	18.9%	28.0%
**European Region**	**24,270**	**22,560**	**21,890**	**21,200**		**1,710**	**2,380**	**3,070**		**7.0%**	**9.8%**	**12.7%**
Azerbaijan	2,790	2,590	2,540	2,460		200	250	330		7.2%	9.0%	11.8%
Kyrgyzstan	1,650	1,590	1,550	1,520		60	100	130		3.6%	6.1%	7.9%
Tajikistan	5,140	4,760	4,590	4,390		380	550	750		7.4%	10.7%	14.6%
Turkmenistan	2,300	2,180	2,120	2,050		120	180	250		5.2%	7.8%	10.9%
Uzbekistan	12,390	11,450	11,100	10,780		940	1,290	1,610		7.6%	10.4%	13.0%
**Eastern Mediterranean Region**	**418,930**	**370,680**	**336,380**	**301,460**		**48,250**	**82,550**	**117,470**		**11.5%**	**19.7%**	**28.0%**
Afghanistan	37,190	30,860	26,670	23,190		6,330	10,520	14,000		17.0%	28.3%	37.6%
Djibouti	690	560	550	540		130	140	150		18.9%	20.3%	21.8%
Egypt	29,670	28,170	27,580	26,950		1,500	2,090	2,720		5.1%	7.0%	9.2%

	Number of neonatal deaths at baseline and at various levels of population coverage in 2020					Number of neonatal deaths prevented at various levels of population coverage				Percentage of neonatal deaths prevented at various levels of population coverage		
**Region/Country**	**No change**	**Target 50**	**Target 70**	**Target 90**		**Target 50**	**Target 70**	**Target 90**		**Target 50**	**Target 70**	**Target 90**
Iraq	24,050	21,800	21,170	20,550		2,250	2,880	3,500		9.4%	12.0%	14.6%
Morocco	11,190	9,980	9,630	8,820		1,210	1,560	2,370		10.8%	13.9%	21.2%
Pakistan	238,680	212,540	190,320	167,620		26,140	48,360	71,060		11.0%	20.3%	29.8%
Somalia	20,100	16,480	14,350	12,270		3,620	5,750	7,830		18.0%	28.6%	39.0%
Sudan	38,650	34,140	31,800	28,750		4,510	6,850	9,900		11.7%	17.7%	25.6%
Yemen	18,720	16,150	14,300	12,770		2,570	4,420	5,950		13.7%	23.6%	31.8%
**Western Pacific Region**	**61,670**	**56,590**	**54,240**	**50,900**		**5,080**	**7,430**	**10,770**		**8.2%**	**12.0%**	**17.5%**
Cambodia	5,310	5,000	4,910	4,720		310	400	590		5.8%	7.5%	11.1%
Lao People's Democratic Republic	5,190	4,430	3,920	3,510		760	1,270	1,680		14.7%	24.5%	32.4%
Papua New Guinea	5,430	4,750	4,300	3,860		680	1,130	1,570		12.5%	20.8%	28.9%
Philippines	28,950	26,320	25,300	23,270		2,630	3,650	5,680		9.1%	12.6%	19.6%
Solomon Islands	200	190	180	170		10	20	30		4.9%	9.8%	14.7%
Viet Nam	16,600	15,900	15,630	15,370		700	970	1,230		4.2%	5.8%	7.4%
**Grand Total**	**2,453,990**	**2,142,620**	**1,943,560**	**1,749,760**		**311,370**	**510,430**	**704,230**		**12.7%**	**20.8%**	**28.7%**

Table 6. Estimates of numbers and percentages of child (1-59 months) deaths that would be saved by expanded coverage of community-based interventions, by country and WHO region

	Number of child (1-59 mo) deaths at baseline and at various levels of population coverage in 2020				Number of child (1-59 mo) deaths prevented at various levels of population coverage			Percentage of child (1-59 mo) deaths prevented at various levels of population coverage		
**Region/Country**	**No change**	**Target 50**	**Target 70**	**Target 90**	**Target 50**	**Target 70**	**Target 90**	**Target 50**	**Target 70**	**Target 90**
**African Region**	**1,929,210**	**1,546,040**	**1,308,250**	**1,064,720**	**383,170**	**620,960**	**864,490**	**19.9%**	**32.2%**	**44.8%**
Angola	129,390	108,090	93,770	78,210	21,300	35,620	51,180	16.5%	27.5%	39.6%
Benin	26,430	20,960	17,680	14,280	5,470	8,750	12,150	20.7%	33.1%	46.0%
Botswana	1,040	910	840	780	130	200	260	12.5%	19.2%	25.0%
Burkina Faso	45,140	34,780	28,890	23,590	10,360	16,250	21,550	23.0%	36.0%	47.7%
Burundi	27,260	23,680	20,820	18,010	3,580	6,440	9,250	13.1%	23.6%	33.9%
Cameroon	51,730	40,490	35,270	30,280	11,240	16,460	21,450	21.7%	31.8%	41.5%
Central African Republic	14,190	10,340	8,100	6,380	3,850	6,090	7,810	27.1%	42.9%	55.0%
Chad	65,180	47,140	38,700	32,090	18,040	26,480	33,090	27.7%	40.6%	50.8%
Comoros	1,050	820	690	570	230	360	480	22.0%	34.4%	45.9%
Congo	4,490	3,770	3,360	2,970	720	1,130	1,520	16.0%	25.1%	33.8%
Côte d'Ivoire	44,940	32,520	26,470	20,300	12,420	18,470	24,640	27.6%	41.1%	54.8%
Democratic Republic of the Congo	225,040	181,200	153,230	123,080	43,840	71,810	101,960	19.5%	31.9%	45.3%
Equatorial Guinea	1,890	1,540	1,270	1,020	350	620	870	18.5%	32.8%	46.0%
Eritrea	4,840	4,110	3,570	3,150	730	1,270	1,690	15.1%	26.3%	34.9%
Ethiopia	98,230	81,910	73,090	61,650	16,320	25,140	36,580	16.6%	25.6%	37.2%
Gabon	1,410	1,180	1,070	880	230	340	530	16.3%	24.1%	37.6%
Gambia	3,340	2,980	2,750	2,390	360	590	950	10.8%	17.6%	28.4%
Ghana	27,810	24,010	20,430	16,870	3,800	7,380	10,940	13.7%	26.5%	39.3%
Guinea	29,220	21,290	16,430	12,620	7,930	12,790	16,600	27.1%	43.8%	56.8%

Region/Country	Number of child (1-59 mo) deaths at baseline and at various levels of population coverage in 2020					Number of child (1-59 mo) deaths prevented at various levels of population coverage				Percentage of child (1-59 mo) deaths prevented at various levels of population coverage		
	No change	Target 50	Target 70	Target 90		Target 50	Target 70	Target 90		Target 50	Target 70	Target 90
Guinea-Bissau	3,500	2,710	2,330	1,960		790	1,170	1,540		22.6%	33.4%	44.0%
Kenya	43,700	38,930	34,980	29,150		4,770	8,720	14,550		10.9%	20.0%	33.3%
Lesotho	3,230	2,880	2,590	2,250		350	640	980		10.8%	19.8%	30.3%
Liberia	7,260	6,160	4,910	3,820		1,100	2,350	3,440		15.2%	32.4%	47.4%
Madagascar	25,530	21,050	18,480	15,480		4,480	7,050	10,050		17.5%	27.6%	39.4%
Malawi	30,230	27,490	25,330	21,390		2,740	4,900	8,840		9.1%	16.2%	29.2%
Mali	59,250	42,430	34,150	25,800		16,820	25,100	33,450		28.4%	42.4%	56.5%
Mauritania	6,570	5,380	4,740	4,140		1,190	1,830	2,430		18.1%	27.9%	37.0%
Mozambique	57,640	48,630	40,510	31,970		9,010	17,130	25,670		15.6%	29.7%	44.5%
Niger	74,470	61,080	50,360	39,320		13,390	24,110	35,150		18.0%	32.4%	47.2%
Nigeria	523,910	393,070	314,950	246,960		130,840	208,960	276,950		25.0%	39.9%	52.9%
Rwanda	8,320	7,340	6,590	5,930		980	1,730	2,390		11.8%	20.8%	28.7%
São Tomé and Príncipe	220	170	150	120		50	70	100		23.3%	32.6%	46.5%
Senegal	15,130	12,910	11,480	10,090		2,220	3,650	5,040		14.7%	24.1%	33.3%
Sierra Leone	19,340	16,450	14,300	10,930		2,890	5,040	8,410		14.9%	26.1%	43.5%
South Africa	31,460	28,970	27,640	24,050		2,490	3,820	7,410		7.9%	12.1%	23.6%
South Sudan	24,560	18,910	15,220	12,360		5,650	9,340	12,200		23.0%	38.0%	49.7%
Swaziland	1,480	1,380	1,260	1,120		100	220	360		6.8%	14.9%	24.3%
Togo	12,920	9,950	8,130	6,460		2,970	4,790	6,460		23.0%	37.1%	50.0%
Uganda	63,260	56,500	50,530	41,850		6,760	12,730	21,410		10.7%	20.1%	33.8%
United Republic of Tanzania	63,040	56,160	51,670	45,110		6,880	11,370	17,930		10.9%	18.0%	28.4%
Zambia	28,150	24,850	22,780	18,690		3,300	5,370	9,460		11.7%	19.1%	33.6%
Zimbabwe	23,460	20,930	18,720	16,690		2,530	4,740	6,770		10.8%	20.2%	28.9%
**Region of the Americas**	**49,380**	**45,920**	**41,830**	**38,170**		**3,460**	**7,550**	**11,210**		**7.0%**	**15.3%**	**22.7%**

	Number of child (1-59 mo) deaths at baseline and at various levels of population coverage in 2020					Number of child (1-59 mo) deaths prevented at various levels of population coverage				Percentage of child (1-59 mo) deaths prevented at various levels of population coverage		
**Region/Country**	**No change**	**Target 50**	**Target 70**	**Target 90**		**Target 50**	**Target 70**	**Target 90**		**Target 50**	**Target 70**	**Target 90**
Bolivia	4,700	4,390	3,980	3,630		310	720	1,070		6.6%	15.3%	22.8%
Brazil	21,670	20,990	19,830	18,830		680	1,840	2,840		3.1%	8.5%	13.1%
Guatemala	6,870	6,280	5,550	4,820		590	1,320	2,050		8.6%	19.2%	29.9%
Haiti	11,030	9,420	7,840	6,580		1,610	3,190	4,450		14.6%	28.9%	40.4%
Peru	5,120	4,850	4,630	4,310		270	490	810		5.3%	9.6%	15.8%
**South-East Asia Region**	**643,540**	**515,210**	**460,120**	**385,490**		**128,330**	**183,420**	**258,050**		**19.9%**	**28.5%**	**40.1%**
Bangladesh	42,230	36,110	32,250	28,010		6,120	9,980	14,220		14.5%	23.6%	33.7%
Dem. People's Republic of Korea	3,950	3,640	3,510	3,160		310	440	790		7.8%	11.1%	20.0%
India	502,670	393,830	349,790	290,970		108,840	152,880	211,700		21.7%	30.4%	42.1%
Indonesia	65,260	55,750	50,880	43,350		9,510	14,380	21,910		14.6%	22.0%	33.6%
Myanmar	21,780	19,040	17,750	14,780		2,740	4,030	7,000		12.6%	18.5%	32.1%
Nepal	7,650	6,840	5,930	5,220		810	1,720	2,430		10.6%	22.5%	31.8%
**European Region**	**24,180**	**21,780**	**20,190**	**18,060**		**2,400**	**3,990**	**6,120**		**9.9%**	**16.5%**	**25.3%**
Azerbaijan	2,130	1,840	1,690	1,550		290	440	580		13.6%	20.7%	27.2%
Kyrgyzstan	1,430	1,310	1,210	1,100		120	220	330		8.4%	15.4%	23.1%
Tajikistan	6,120	5,520	5,060	4,430		600	1,060	1,690		9.8%	17.3%	27.6%
Turkmenistan	2,960	2,720	2,450	2,210		240	510	750		8.1%	17.2%	25.3%
Uzbekistan	11,540	10,390	9,780	8,780		1,150	1,760	2,760		10.0%	15.2%	23.9%
**Eastern Mediterranean Region**	**409,730**	**352,640**	**319,610**	**275,420**		**57,090**	**90,120**	**134,310**		**13.9%**	**22.0%**	**32.8%**
Afghanistan	57,690	51,840	45,200	36,960		5,850	12,490	20,730		10.1%	21.6%	35.9%
Djibouti	650	580	530	450		70	120	200		10.7%	18.4%	30.7%
Egypt	25,970	23,750	22,600	20,760		2,220	3,370	5,210		8.5%	13.0%	20.1%
Iraq	17,330	15,300	14,380	12,580		2,030	2,950	4,750		11.7%	17.0%	27.4%

	Number of child (1-59 mo) deaths at baseline and at various levels of population coverage in 2020					Number of child (1-59 mo) deaths prevented at various levels of population coverage				Percentage of child (1-59 mo) deaths prevented at various levels of population coverage		
**Region/Country**	**No change**	**Target 50**	**Target 70**	**Target 90**		**Target 50**	**Target 70**	**Target 90**		**Target 50**	**Target 70**	**Target 90**
Morocco	6,420	5,890	5,660	5,120		530	760	1,300		8.3%	11.8%	20.2%
Pakistan	186,680	162,990	151,380	129,820		23,690	35,300	56,860		12.7%	18.9%	30.5%
Somalia	47,210	33,890	28,080	23,480		13,320	19,130	23,730		28.2%	40.5%	50.3%
Sudan	51,150	44,070	38,920	34,740		7,080	12,230	16,410		13.8%	23.9%	32.1%
Yemen	16,640	14,350	12,860	11,520		2,290	3,780	5,120		13.8%	22.7%	30.8%
**Western Pacific Region**	**69,020**	**61,170**	**55,720**	**48,040**		**7,850**	**13,300**	**20,980**		**11.4%**	**19.3%**	**30.4%**
Cambodia	5,030	4,440	4,150	3,620		590	880	1,410		11.7%	17.5%	28.0%
Lao People's Democratic Republic	6,250	5,540	4,890	4,230		710	1,360	2,020		11.4%	21.8%	32.3%
Papua New Guinea	7,150	5,500	4,700	3,760		1,650	2,450	3,390		23.1%	34.3%	47.4%
Philippines	35,200	31,730	28,830	24,190		3,470	6,370	11,010		9.9%	18.1%	31.3%
Solomon Islands	270	210	190	170		60	80	100		22.6%	30.2%	37.7%
Viet Nam	15,130	13,760	12,970	12,070		1,370	2,160	3,060		9.1%	14.3%	20.2%
**Grand Total**	**3,125,070**	**2,542,760**	**2,205,720**	**1,829,900**		**582,310**	**919,350**	**1,295,170**		**18.6%**	**29.4%**	**41.4%**

Table 7. Estimates of numbers and percentages of deaths of all under-five children (0-59 months) that would be saved by expanded coverage of community-based interventions, by country and WHO region

	**Number of total child (0-59 mo) deaths at baseline and at various levels of population coverage in 2020**					**Number of total child (0-59 mo) deaths prevented at various levels of population coverage**				**Percentage of total child (0-59 mo) deaths prevented at various levels of population coverage**		
**Region/Country**	**No change**	**Target 50**	**Target 70**	**Target 90**		**Target 50**	**Target 70**	**Target 90**		**Target 50**	**Target 70**	**Target 90**
**African Region**	**2,964,150**	**2,435,440**	**2,115,220**	**1,785,330**		**528,710**	**848,930**	**1,178,820**		**17.8%**	**28.6%**	**39.8%**
Angola	189,830	160,960	140,330	119,190		28,870	49,500	70,640		15.2%	26.1%	37.2%
Benin	39,100	32,400	28,650	24,190		6,700	10,450	14,910		17.1%	26.7%	38.1%
Botswana	2,160	1,970	1,870	1,790		190	290	370		8.8%	13.5%	17.2%
Burkina Faso	65,260	52,310	45,610	38,540		12,950	19,650	26,720		19.8%	30.1%	40.9%
Burundi	42,310	37,000	33,470	29,360		5,310	8,840	12,950		12.5%	20.9%	30.6%
Cameroon	73,910	60,520	54,300	47,100		13,390	19,610	26,810		18.1%	26.5%	36.3%
Central African Republic	21,210	16,410	13,490	11,100		4,800	7,720	10,110		22.6%	36.4%	47.7%
Chad	91,700	68,070	56,890	47,880		23,630	34,810	43,820		25.8%	38.0%	47.8%
Comoros	1,950	1,640	1,480	1,320		310	470	630		15.9%	24.1%	32.3%
Congo	7,610	6,690	6,200	5,710		920	1,410	1,900		12.1%	18.5%	25.0%
Côte d'Ivoire	76,870	60,600	52,380	43,300		16,270	24,490	33,570		21.2%	31.9%	43.7%
Democratic Republic of the Congo	327,020	273,200	241,890	205,280		53,820	85,130	121,740		16.5%	26.0%	37.2%
Equatorial Guinea	2,960	2,520	2,210	1,870		440	750	1,090		14.9%	25.3%	36.8%
Eritrea	8,030	6,780	5,930	5,270		1,250	2,100	2,760		15.6%	26.2%	34.4%
Ethiopia	188,030	151,710	134,310	115,170		36,320	53,720	72,860		19.3%	28.6%	38.7%
Gabon	2,600	2,270	2,130	1,910		330	470	690		12.7%	18.1%	26.6%
Gambia	6,010	5,310	4,880	4,290		700	1,130	1,720		11.6%	18.8%	28.6%

Region/Country	Number of total child (0-59 mo) deaths at baseline and at various levels of population coverage in 2020					Number of total child (0-59 mo) deaths prevented at various levels of population coverage				Percentage of total child (0-59 mo) deaths prevented at various levels of population coverage		
	No change	Target 50	Target 70	Target 90		Target 50	Target 70	Target 90		Target 50	Target 70	Target 90
Ghana	52,070	46,110	41,780	36,020		5,960	10,290	16,050		11.4%	19.8%	30.8%
Guinea	44,160	34,070	27,600	22,500		10,090	16,560	21,660		22.9%	37.5%	49.1%
Guinea-Bissau	6,190	5,010	4,340	3,720		1,180	1,850	2,470		19.1%	29.9%	39.9%
Kenya	79,650	71,380	65,240	56,150		8,270	14,410	23,500		10.4%	18.1%	29.5%
Lesotho	5,220	4,650	4,240	3,720		570	980	1,500		10.9%	18.8%	28.7%
Liberia	11,120	9,580	8,080	6,610		1,540	3,040	4,510		13.9%	27.3%	40.6%
Madagascar	42,900	35,840	31,470	27,050		7,060	11,430	15,850		16.5%	26.6%	36.9%
Malawi	46,630	42,930	40,410	35,800		3,700	6,220	10,830		7.9%	13.3%	23.2%
Mali	89,480	68,190	57,650	46,430		21,290	31,830	43,050		23.8%	35.6%	48.1%
Mauritania	11,490	9,650	8,780	7,760		1,840	2,710	3,730		16.0%	23.6%	32.5%
Mozambique	89,610	75,750	64,870	53,490		13,860	24,740	36,120		15.5%	27.6%	40.3%
Niger	105,120	86,100	72,110	58,490		19,020	33,010	46,630		18.1%	31.4%	44.4%
Nigeria	774,090	603,550	498,230	408,940		170,540	275,860	365,150		22.0%	35.6%	47.2%
Rwanda	15,010	13,290	12,360	11,260		1,720	2,650	3,750		11.5%	17.7%	25.0%
São Tomé and Príncipe	340	290	270	240		50	70	100		14.7%	20.6%	29.5%
Senegal	27,240	23,740	21,420	18,930		3,500	5,820	8,310		12.9%	21.4%	30.5%
Sierra Leone	27,220	23,380	20,660	16,510		3,840	6,560	10,710		14.1%	24.1%	39.3%
South Africa	43,160	39,860	38,240	34,370		3,300	4,920	8,790		7.6%	11.4%	20.4%
South Sudan	43,010	33,500	27,840	23,120		9,510	15,170	19,890		22.1%	35.3%	46.2%
Swaziland	1,960	1,830	1,700	1,540		130	260	420		6.6%	13.3%	21.5%
Togo	19,770	16,110	13,800	11,440		3,660	5,970	8,330		18.5%	30.2%	42.1%
Uganda	97,810	87,040	78,830	67,030		10,770	18,980	30,780		11.0%	19.4%	31.5%
United Republic of Tanzania	104,740	92,310	83,800	73,860		12,430	20,940	30,880		11.9%	20.0%	29.5%
Zambia	43,770	38,760	35,920	30,370		5,010	7,850	13,400		11.4%	17.9%	30.6%

Region/Country	Number of total child (0-59 mo) deaths at baseline and at various levels of population coverage in 2020					Number of total child (0-59 mo) deaths prevented at various levels of population coverage				Percentage of total child (0-59 mo) deaths prevented at various levels of population coverage		
	No change	Target 50	Target 70	Target 90		Target 50	Target 70	Target 90		Target 50	Target 70	Target 90
Zimbabwe	35,870	32,190	29,580	26,720		3,680	6,290	9,150		10.3%	17.5%	25.5%
**Region of the Americas**	**96,420**	**89,070**	**83,100**	**77,430**		**7,350**	**13,320**	**18,990**		**7.6%**	**13.8%**	**19.7%**
Bolivia	9,610	8,970	8,480	7,750		640	1,130	1,860		6.7%	11.8%	19.3%
Brazil	46,730	44,400	42,600	41,010		2,330	4,130	5,720		5.0%	8.8%	12.2%
Guatemala	12,710	11,450	10,300	9,160		1,260	2,410	3,550		9.9%	19.0%	27.9%
Haiti	17,460	14,820	12,560	10,770		2,640	4,900	6,690		15.1%	28.1%	38.3%
Peru	9,900	9,440	9,160	8,740		460	740	1,160		4.6%	7.5%	11.7%
**South-East Asia Region**	**1,510,680**	**1,275,450**	**1,142,940**	**1,001,820**		**235,230**	**367,740**	**508,860**		**15.6%**	**24.3%**	**33.7%**
Bangladesh	109,910	94,200	83,160	73,170		15,710	26,750	36,740		14.3%	24.3%	33.4%
Dem. People's Republic of Korea	8,690	8,000	7,740	7,250		690	950	1,440		7.9%	10.9%	16.6%
India	1,196,670	1,000,590	890,630	777,190		196,080	306,040	419,480		16.4%	25.6%	35.1%
Indonesia	128,980	113,970	107,000	96,600		15,010	21,980	32,380		11.6%	17.0%	25.1%
Myanmar	46,360	40,770	38,400	33,460		5,590	7,960	12,900		12.1%	17.2%	27.8%
Nepal	20,070	17,920	16,010	14,160		2,150	4,060	5,910		10.7%	20.2%	29.4%
**European Region**	**48,450**	**44,340**	**42,080**	**39,260**		**4,110**	**6,370**	**9,190**		**8.5%**	**13.1%**	**19.0%**
Azerbaijan	4,920	4,430	4,220	4,010		490	700	910		10.0%	14.2%	18.5%
Kyrgyzstan	3,080	2,890	2,760	2,610		190	320	470		6.2%	10.4%	15.3%
Tajikistan	11,260	10,280	9,650	8,820		980	1,610	2,440		8.7%	14.3%	21.7%
Turkmenistan	5,270	4,900	4,560	4,250		370	710	1,020		7.0%	13.5%	19.4%
Uzbekistan	23,930	21,840	20,880	19,560		2,090	3,050	4,370		8.7%	12.7%	18.3%
**Eastern Mediterranean Region**	**828,670**	**723,320**	**655,990**	**576,880**		**105,350**	**172,680**	**251,790**		**12.7%**	**20.8%**	**30.4%**
Afghanistan	94,880	82,700	71,870	60,150		12,180	23,010	34,730		12.8%	24.3%	36.6%

Region/Country	Number of total child (0-59 mo) deaths at baseline and at various levels of population coverage in 2020					Number of total child (0-59 mo) deaths prevented at various levels of population coverage				Percentage of total child (0-59 mo) deaths prevented at various levels of population coverage		
	No change	Target 50	Target 70	Target 90		Target 50	Target 70	Target 90		Target 50	Target 70	Target 90
Djibouti	1,340	1,140	1,080	990		200	260	350		14.9%	19.4%	26.1%
Egypt	55,640	51,910	50,180	47,710		3,730	5,460	7,930		6.7%	9.8%	14.3%
Iraq	41,380	37,100	35,550	33,120		4,280	5,830	8,260		10.3%	14.1%	20.0%
Morocco	17,620	15,860	15,290	13,940		1,760	2,330	3,680		10.0%	13.2%	20.9%
Pakistan	425,360	375,540	341,710	297,440		49,820	83,650	127,920		11.7%	19.7%	30.1%
Somalia	67,300	50,370	42,430	35,750		16,930	24,870	31,550		25.2%	37.0%	46.9%
Sudan	89,790	78,210	70,720	63,490		11,580	19,070	26,300		12.9%	21.2%	29.3%
Yemen	35,350	30,490	27,170	24,290		4,860	8,180	11,060		13.7%	23.1%	31.3%
**Western Pacific Region**	**130,690**	**117,760**	**109,960**	**98,940**		**12,930**	**20,730**	**31,750**		**9.9%**	**15.9%**	**24.3%**
Cambodia	10,340	9,430	9,060	8,340		910	1,280	2,000		8.8%	12.4%	19.3%
Lao People's Democratic Republic	11,430	9,970	8,800	7,740		1,460	2,630	3,690		12.8%	23.0%	32.3%
Papua New Guinea	12,590	10,250	9,000	7,610		2,340	3,590	4,980		18.6%	28.5%	39.6%
Philippines	64,140	58,050	54,130	47,470		6,090	10,010	16,670		9.5%	15.6%	26.0%
Solomon Islands	470	400	370	340		70	100	130		14.9%	21.3%	27.7%
Viet Nam	31,720	29,660	28,610	27,440		2,060	3,110	4,280		6.5%	9.8%	13.5%
**Grand Total**	**5,579,060**	**4,685,380**	**4,149,280**	**3,579,650**		**893,680**	**1,429,780**	**1,999,410**		**16.0%**	**25.6%**	**35.8%**

Table 8. Estimates of numbers and percentages of ALL deaths (maternal, child and stillbirths combined) that would be averted by expanded coverage of community-based interventions, by country and WHO region

	Number of total deaths at baseline and at various levels of population coverage in 2020					Number of total deaths prevented at various levels of population coverage				Percentage of total deaths prevented at various levels of population coverage		
**Region/Country**	**No change**	**Target 50**	**Target 70**	**Target 90**		**Target 50**	**Target 70**	**Target 90**		**Target 50**	**Target 70**	**Target 90**
**African Region**	**4,210,940**	**3,502,950**	**3,097,760**	**2,680,410**		**707,990**	**1,113,180**	**1,530,530**		**16.8%**	**26.4%**	**36.3%**
Angola	229,610	197,690	174,850	151,570		31,920	54,760	78,040		13.9%	23.8%	34.0%
Benin	52,790	44,010	39,390	33,870		8,780	13,400	18,920		16.6%	25.4%	35.8%
Botswana	3,000	2,750	2,630	2,520		250	370	480		8.3%	12.3%	16.0%
Burkina Faso	83,970	68,010	60,160	51,590		15,960	23,810	32,380		19.0%	28.4%	38.6%
Burundi	60,030	51,900	47,200	41,710		8,130	12,830	18,320		13.5%	21.4%	30.5%
Cameroon	96,080	80,460	73,070	64,340		15,620	23,010	31,740		16.3%	23.9%	33.0%
Central African Republic	28,350	22,430	18,890	15,910		5,920	9,460	12,440		20.9%	33.4%	43.9%
Chad	124,390	95,990	82,640	71,570		28,400	41,750	52,820		22.8%	33.6%	42.5%
Comoros	2,860	2,480	2,300	2,100		380	560	760		13.3%	19.6%	26.6%
Congo	10,980	9,720	9,080	8,450		1,260	1,900	2,530		11.5%	17.3%	23.0%
Côte d'Ivoire	104,860	85,530	75,710	64,740		19,330	29,150	40,120		18.4%	27.8%	38.3%
Democratic Republic of the Congo	443,130	368,470	329,100	283,590		74,660	114,030	159,540		16.8%	25.7%	36.0%
Equatorial Guinea	3,600	3,120	2,810	2,440		480	790	1,160		13.4%	22.0%	32.3%
Eritrea	12,810	10,940	9,790	8,860		1,870	3,020	3,950		14.6%	23.6%	30.8%
Ethiopia	295,890	244,860	221,550	196,750		51,030	74,340	99,140		17.2%	25.1%	33.5%
Gabon	3,470	3,060	2,880	2,620		410	590	850		11.8%	17.0%	24.5%
Gambia	8,760	7,770	7,170	6,370		990	1,590	2,390		11.3%	18.1%	27.3%
Ghana	74,290	66,480	61,370	53,790		7,810	12,920	20,500		10.5%	17.4%	27.6%

Region/Country	Number of total deaths at baseline and at various levels of population coverage in 2020					Number of total deaths prevented at various levels of population coverage				Percentage of total deaths prevented at various levels of population coverage		
	No change	Target 50	Target 70	Target 90		Target 50	Target 70	Target 90		Target 50	Target 70	Target 90
Guinea	57,440	45,780	38,420	32,470		11,660	19,020	24,970		20.3%	33.1%	43.5%
Guinea-Bissau	9,050	7,350	6,430	5,580		1,700	2,620	3,470		18.8%	28.9%	38.3%
Kenya	124,340	111,020	102,320	90,170		13,320	22,020	34,170		10.7%	17.7%	27.5%
Lesotho	6,700	5,940	5,440	4,820		760	1,260	1,880		11.3%	18.8%	28.1%
Liberia	15,740	13,570	11,730	9,870		2,170	4,010	5,870		13.8%	25.5%	37.3%
Madagascar	62,100	51,160	45,000	38,940		10,940	17,100	23,160		17.6%	27.5%	37.3%
Malawi	67,850	61,080	57,150	51,030		6,770	10,700	16,820		10.0%	15.8%	24.8%
Mali	120,150	94,470	81,810	68,220		25,680	38,340	51,930		21.4%	31.9%	43.2%
Mauritania	16,080	13,960	12,960	11,720		2,120	3,120	4,360		13.2%	19.4%	27.1%
Mozambique	117,970	99,400	86,210	72,520		18,570	31,760	45,450		15.7%	26.9%	38.5%
Niger	153,370	126,970	109,290	92,230		26,400	44,080	61,140		17.2%	28.7%	39.9%
Nigeria	1,147,270	917,130	783,080	666,960		230,140	364,190	480,310		20.1%	31.7%	41.9%
Rwanda	22,220	19,370	17,980	16,350		2,850	4,240	5,870		12.8%	19.1%	26.4%
São Tomé and Príncipe	470	410	380	340		60	90	130		12.8%	19.2%	27.8%
Senegal	43,350	37,980	34,530	30,790		5,370	8,820	12,560		12.4%	20.3%	29.0%
Sierra Leone	35,860	31,130	27,880	23,110		4,730	7,980	12,750		13.2%	22.3%	35.6%
South Africa	63,100	58,370	56,180	51,740		4,730	6,920	11,360		7.5%	11.0%	18.0%
South Sudan	60,900	48,620	41,770	35,940		12,280	19,130	24,960		20.2%	31.4%	41.0%
Swaziland	2,500	2,320	2,170	1,990		180	330	510		7.2%	13.2%	20.4%
Togo	29,500	24,450	21,360	18,150		5,050	8,140	11,350		17.1%	27.6%	38.5%
Uganda	143,050	127,060	115,980	100,970		15,990	27,070	42,080		11.2%	18.9%	29.4%
United Republic of Tanzania	163,320	143,070	129,690	115,200		20,250	33,630	48,120		12.4%	20.6%	29.5%
Zambia	60,650	53,030	49,060	41,860		7,620	11,590	18,790		12.6%	19.1%	31.0%
Zimbabwe	49,110	43,660	40,360	36,660		5,450	8,750	12,450		11.1%	17.8%	25.4%

	Number of total deaths at baseline and at various levels of population coverage in 2020				Number of total deaths prevented at various levels of population coverage			Percentage of total deaths prevented at various levels of population coverage		
**Region/Country**	**No change**	**Target 50**	**Target 70**	**Target 90**	**Target 50**	**Target 70**	**Target 90**	**Target 50**	**Target 70**	**Target 90**
**Region of the Americas**	**144,080**	**133,550**	**126,260**	**119,230**	**10,530**	**17,820**	**24,850**	**7.3%**	**12.4%**	**17.2%**
Bolivia	13,370	12,520	11,950	11,120	850	1,420	2,250	6.4%	10.6%	16.8%
Brazil	72,170	68,500	66,170	64,030	3,670	6,000	8,140	5.1%	8.3%	11.3%
Guatemala	18,280	16,660	15,340	14,020	1,620	2,940	4,260	8.9%	16.1%	23.3%
Haiti	24,700	21,060	18,380	16,180	3,640	6,320	8,520	14.7%	25.6%	34.5%
Peru	15,550	14,810	14,420	13,890	740	1,130	1,660	4.8%	7.3%	10.7%
**South-East Asia Region**	**2,315,080**	**2,012,050**	**1,838,610**	**1,655,810**	**303,030**	**476,470**	**659,270**	**13.1%**	**20.6%**	**28.5%**
Bangladesh	188,960	163,150	146,920	132,010	25,810	42,040	56,950	13.7%	22.2%	30.1%
Dem. People's Republic of Korea	13,740	12,400	11,890	11,160	1,340	1,850	2,580	9.8%	13.5%	18.8%
India	1,816,600	1,570,580	1,428,110	1,281,740	246,020	388,490	534,860	13.5%	21.4%	29.4%
Indonesia	197,250	177,460	168,630	156,080	19,790	28,620	41,170	10.0%	14.5%	20.9%
Myanmar	66,670	59,550	56,570	50,790	7,120	10,100	15,880	10.7%	15.1%	23.8%
Nepal	31,860	28,920	26,480	24,040	2,940	5,380	7,820	9.2%	16.9%	24.5%
**European Region**	**65,450**	**59,460**	**56,480**	**52,950**	**5,990**	**8,970**	**12,500**	**9.2%**	**13.7%**	**19.1%**
Azerbaijan	7,490	6,870	6,610	6,350	620	880	1,140	8.3%	11.7%	15.2%
Kyrgyzstan	4,650	4,390	4,230	4,050	260	420	600	5.6%	9.0%	12.9%
Tajikistan	14,840	13,670	12,970	12,060	1,170	1,870	2,780	7.9%	12.6%	18.7%
Turkmenistan	7,040	6,450	6,030	5,640	590	1,010	1,400	8.4%	14.3%	19.9%
Uzbekistan	31,430	28,070	26,640	24,840	3,360	4,790	6,590	10.7%	15.2%	21.0%
**Eastern Mediterranean Region**	**1,248,680**	**1,112,660**	**1,028,610**	**930,900**	**136,020**	**220,070**	**317,780**	**10.9%**	**17.6%**	**25.4%**
Afghanistan	127,050	110,500	97,620	83,960	16,550	29,430	43,090	13.0%	23.2%	33.9%

Region/Country	Number of total deaths at baseline and at various levels of population coverage in 2020					Number of total deaths prevented at various levels of population coverage				Percentage of total deaths prevented at various levels of population coverage		
	No change	Target 50	Target 70	Target 90		Target 50	Target 70	Target 90		Target 50	Target 70	Target 90
Djibouti	2,100	1,840	1,760	1,640		260	340	460		12.4%	16.2%	21.9%
Egypt	84,680	78,660	76,030	72,670		6,020	8,650	12,010		7.1%	10.2%	14.2%
Iraq	62,290	56,870	54,870	51,990		5,420	7,420	10,300		8.7%	11.9%	16.5%
Morocco	33,980	31,410	30,500	28,680		2,570	3,480	5,300		7.6%	10.2%	15.6%
Pakistan	660,910	597,140	553,830	498,540		63,770	107,080	162,370		9.6%	16.2%	24.6%
Somalia	89,010	68,540	59,060	50,940		20,470	29,950	38,070		23.0%	33.6%	42.8%
Sudan	125,480	111,500	103,070	94,470		13,980	22,410	31,010		11.1%	17.9%	24.7%
Yemen	63,180	56,210	51,880	48,020		6,970	11,300	15,160		11.0%	17.9%	24.0%
**Western Pacific Region**	**187,440**	**170,740**	**161,360**	**148,440**		**16,700**	**26,080**	**39,000**		**8.9%**	**13.9%**	**20.8%**
Cambodia	15,180	14,030	13,550	12,720		1,150	1,630	2,460		7.6%	10.7%	16.2%
Lao People's Democratic Republic	15,860	13,950	12,560	11,290		1,910	3,300	4,570		12.0%	20.8%	28.8%
Papua New Guinea	16,590	13,810	12,330	10,730		2,780	4,260	5,860		16.8%	25.7%	35.3%
Philippines	91,800	83,900	79,270	71,580		7,900	12,530	20,220		8.6%	13.6%	22.0%
Solomon Islands	780	660	620	570		120	160	210		15.3%	20.5%	26.9%
Viet Nam	47,230	44,390	43,030	41,560		2,840	4,200	5,670		6.0%	8.9%	12.0%
**Grand Total**	**8,171,660**	**6,991,400**	**6,309,070**	**5,587,750**		**1,180,260**	**1,862,590**	**2,583,910**		**14.4%**	**22.8%**	**31.6%**

For more details about the Lives Saved Tool, information and additional resources are available:

http://www.livessavedtool.org/

LiST
